# Long-Term Starvation and Posterior Feeding Effects on Biochemical and Physiological Responses of Midgut Gland of *Cherax quadricarinatus* Juveniles (Parastacidae)

**DOI:** 10.1371/journal.pone.0150854

**Published:** 2016-03-28

**Authors:** Hernán Javier Sacristán, Martín Ansaldo, Luis Marcelo Franco-Tadic, Analía Verónica Fernández Gimenez, Laura Susana López Greco

**Affiliations:** 1 Biology of Reproduction and Growth in Crustaceans, Department of Biodiversity and Experimental Biology, Buenos Aires University, Buenos Aires, Argentina; 2 IBBEA, CONICET-UBA, Buenos Aires, Argentina; 3 Ecophysiology and Ecotoxicology Laboratory, Argentinian Antarctic Institute, Buenos Aires, Argentina; 4 Physiology of Aquatic Organisms, Institute of Marine and Coastal Research, CONICET, Mar del Plata University, Mar del Plata, Argentina; Sonoma State University, UNITED STATES

## Abstract

We investigated the effect of long-term starvation and posterior feeding on energetic reserves, oxidative stress, digestive enzymes, and histology of *C*. *quadricarinatus* midgut gland. The crayfish (6.27 g) were randomly assigned to one of three feeding protocols: continuous feeding throughout 80 day, continuous starvation until 80 day, and continuous starvation throughout 50 day and then feeding for the following 30 days. Juveniles from each protocol were weighed, and sacrificed at day 15, 30, 50 or 80. The lipids, glycogen, reduced glutathione (GSH), soluble protein, lipid peroxidation (TBARS), protein oxidation (PO), catalase (CAT), lipase and proteinase activities, and histology were measured on midgut gland. Starved crayfish had a lower hepatosomatic index, number of molts, specific growth rate, lipids, glycogen, and GSH levels than fed animals at all assay times. The starvation did not affect the soluble protein, TBARS, PO levels and CAT. In starved juveniles the lipase activity decreased as starvation time increased, whereas proteinase activity decreased only at day 80. The histological analysis of the starved animals showed several signs of structural alterations. After 30 days of feeding, the starved-feeding animals exhibited a striking recovery of hepatosomatic index, number of molts, lipids and glycogen, GSH, lipase activity and midgut gland structure.

## Introduction

Crustaceans experience starvation periods during their growing process as a result of sequential molting. Nevertheless, the ability of some crustacean species, such as *Cherax destructor*, to survive during periods of extreme deficiency in food and/or surface water is inevitably linked to its behavioral and physiological response to such conditions. Therefore, survival time is probably associated to nutrient storage capacity and energy conservation [1].

The midgut gland of crustaceans plays a fundamental role during growth and molting, being the principal organ for synthesis and secretion of digestive enzymes, absorption and storage of nutrients such as lipid and glycogen, which can be mobilized during non-feeding periods [2, 3]. The nutrition of the animals can be assessed by the activity of this gland, which is related to several environmental variables, in fact, even starvation has been suggested to be responsible for the reallocation of energy resources for the maintenance of essential aspects of tissue maintenance and survival in spite of the metabolic cost [1, 4–7]. In addition to food deprivation, changes in salinity and food ingredients have been described as modifiers of the midgut gland structure [1, 3, 8–12].

In decapod crustaceans, the digestive enzymes activity does not remain constant during the developmental cycles [13] as a result of internal and external factors such as starvation, availability, quantity and quality of food. Actually, *C quadricarinatus* showed that it can regulate the digestive depending of food availability, Loya-Javellana et al., 1995 [14], and Sacristán et al. (2014) [15]. Furthermore, many studies demonstrated that starvation affects the activity of digestive enzymes [16–21], but the regulation of these enzymes during long-term starvation, is unknown. In particular, Sacristán et al. (2014) [15] demonstrated in *Cherax quadricarinatus*, that the protease activity was not affected by food deprivation, inasmuch as the lipase activity decreased after 16 days of starvation [15].

Recent findings on oxidative stress and antioxidant defenses in crustaceans’ physiology are barely now being revealed. [22]. Cells continuously produce reactive oxygen species (ROS) as a consequence of their reductive oxygen metabolism [23]. All organisms present both enzymatic (superoxide dismutase, catalase and glutathione-S-transferase) and non-enzymatic [reduced glutathione (GSH), and vitamins C and E] antioxidant defense systems against ROS generation. The enzymatic and non-enzymatic defenses act in conjunction to remove or transform ROS into less toxic metabolites [24, 25]. Oxidative stress is produced when the rates of ROS formation overwhelm the antioxidant capacity [26], as a consequence, this increase in ROS exerts damage to different cellular targets [27], such as DNA, lipids and proteins which modify the normal cellular functions [24].

The starved condition has pro-oxidant effects due to the reduction of the antioxidant defense levels [23]. Under this unnatural situation, the protein synthesis was depressed driving the reduction on enzyme levels involved in the ROS neutralization, such as reduced glutathione (GSH) [23]. Although there have been some studies in crabs, crayfish, prawn and shrimp about the effect of food deprivation on many biochemical and physiological variables [3, 15, 19–21, 28–34], little is known about the effects of long-term starvation on oxidative stress parameters and midgut gland functionality.

The redclaw crayfish, *C*. *quadricarinatus*, is a freshwater decapod crustacean, native of North of Queensland (Australia) and Southeast Papua New Guinea. *C*. *quadricarinatus* aquaculture is quite accessible, due to its reproduction efficiency, relative swift growth rate, tolerance to crowding, and malleable eating habits [35–37]. Recent studies on the species reveal a strong starvation resistance in juveniles [12, 38] being capable of undergoing compensatory growth [39, 40].

The aim of the present study was to determine the effect of long-term starvation and posterior feeding on energetic reserves, oxidative stress, digestive enzymes, and histology of the midgut gland in advanced juveniles of *C*. *quadricarinatus*.

## Materials and Methods

### Live specimens

Redclaw crayfish juveniles were hatched from a reproductive female stock supplied by Centro Nacional de Desarrollo Acuícola (CENADAC), Corrientes, Argentina (27° 22’ 42.09”S; 58° 40’ 52.41”O). Each ovigerous female (59.8 ± 3.2 g mean body weight was maintained in an individual glass aquarium (60×40×30 cm; width x length x height). The mean number of eggs carried by females was 100–150. Each aquarium contained 30l of dechlorinated tap water and with continuous aeration (5mg O_2_/l). The temperature was maintained at 27±1°C by ALTMAN water heaters (100W, precision ± 1°C), and the photoperiod cycle was 14 h light: 10 h dark. Each aquarium was provided with a PVC tube´s cave (10 cm in diameter and 25 cm long) [41]. Females were fed daily *ad libitum* with *Elodea* sp. and commercial TetraColor granules TETRA^®^ (47.5% crude protein, 6.5% crude fat, 2.0% crude fiber, 6.0% moisture, 1.5% phosphorus, and 100 mg ascorbic acid/kg) according to Bugnot and López Greco (2009) [42] and Sánchez De Bock and López Greco (2010) [43]. Juveniles became independent at stage 3 [44], then they were separated from their mothers. Thus, they were pooled and maintained to reach the desired experimental weight under conditions described in previous studies [38, 45, 46].

### Experimental design

A total of 109 juveniles (6.27±1.24 g) were placed in individual glass containers (1500 cm^3^) with 1400 mL of filtered water under continuous aeration. They were fed daily *ad libitum* with TetraColor granules (TETRA^®^). In order to maintain the temperature constant at 27±1°C, the containers were placed in aquaria (53×40×12 cm; width x length x height) with water heaters [38]. The photoperiod cycle was set at 14 h light: 10 h dark. Crayfish were acclimated to these experimental conditions for 1 week before the experiment´s onset. The first day of the assay [time 0 (T0)], 10 juveniles were anesthetized in cold water, weighed (precision 0.1 mg) and the midgut gland was dissected and frozen at -80°C.

The remaining animals were randomly distributed in two groups: 43 in the Fed group (F) (control) and 56 in the Starved group (S). The control group was daily fed throughout the 80 days of entire experimental time. The starved animals were not fed until day 50, and thereafter half the animals were starved for the remainder of the experimental period, whereas the other half was fed up to the end of the experiment at day 80 (T80) [Group Starved-fed (SF)]. During the experimental period, the numbers of molts were recorded for each treatment and the containers were cleaned and water was renewed twice a week.

**Fig 1:**
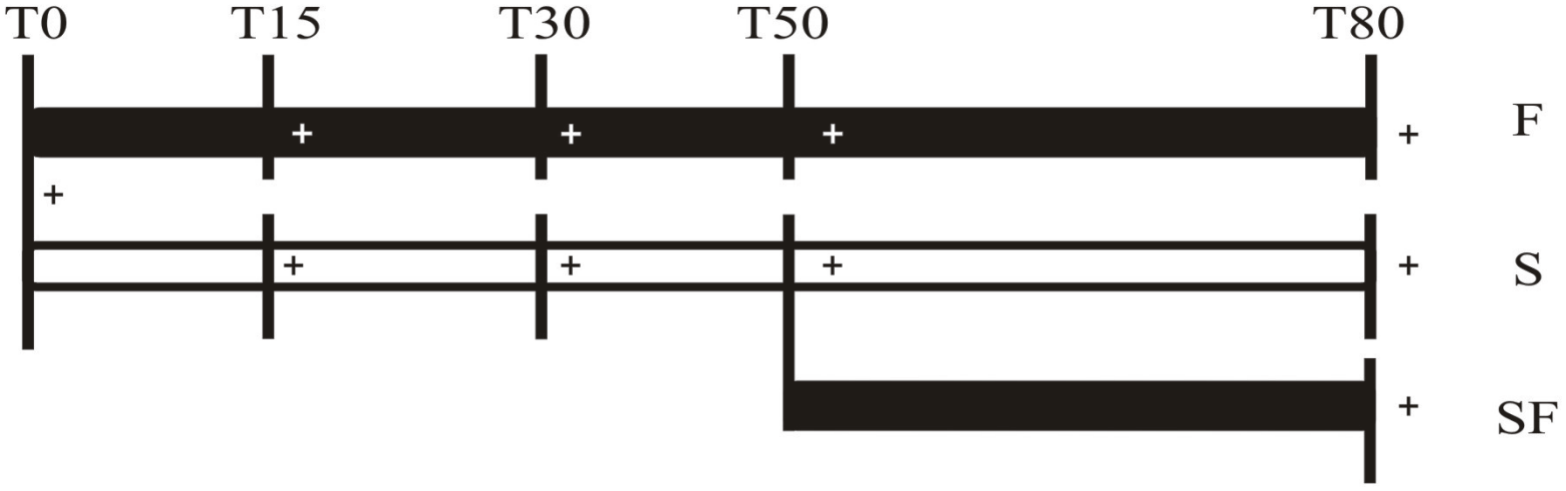
Experimental design scheme used to study the effect of long fasting in *Cherax quadricarinatus* juveniles. Treatments: F: Fed Group; S: Starved Group and SF: Starved-fed Group. T0: end of the acclimation week; T15: 15 days of experiment; T30: 30 days of experiment; T50: 50 days of experiment and T80: 80 days of experiment. The symbol “+” indicate that 10 animals were sacrificed.

At days 15, 30 and 50 (T15, T30, T50 respectively), 10 juveniles of F and S groups, were cold anaesthetized at -20°C for 15 minutes, weighed and each midgut gland dissected and immediately frozen at -80°C. In the same way, 10 animals from the three groups (F, S and SF) were cold anesthetized and sacrificed at the end of the experiment (T80). During experimental phase, the mortality in F and S treatments did not exceed 10% and 20%, respectively. Starvation days were established according to Calvo et al. (2013) [21]. An experimental scheme is shown in Fig 1.

The growth performance was evaluated through the analysis of the specific growth rate (SGR), based on the following equation: SGR (%/day) = [100 x (Ln BWf—Ln BWi)] time (days), where BWf was the final body weight and BWi was the initial body weight (Reynolds 2002) [47]. The hepatosomatic index was determined using the following equation: Hepatosomatic index (HSI) (%) = (midgut gland weight/whole body weight) x 100.

Moreover, for the midgut gland histological analysis at 80 days, 3 juveniles from each group (F, S and SF) were anesthetized and sacrificed. Each midgut gland was fixed in Bouin´s solution and processed with a routine histological method. Briefly, the midgut gland tissues were dehydrated in an alcohol series, embedded in paraffin, and 6 μm sections were stained with hematoxylin-eosin and analyzed under light microscopy [48]. The abnormalities in the mid gland structure were described based on previous studies on the same species [12, 21].

### Energetic reserves, oxidative stress and digestive enzymes activity

A small portion (milligrams) of each midgut gland was used for measuring the reduced glutathione (GSH) levels, lipids and glycogen total reserves. The rest of the organ was utilized for the assessment of soluble protein content, lipid peroxidation (TBARS) and protein oxidation (PO) levels, as well as catalase (CAT), lipase and proteinase activities.

Total lipids were extracted by homogenizing the tissue (40–60 mg) with a mixture of chloroform: methanol (2:1 v/v) described by to Folch et al. (1957) [49]. According to that, the homogenate was filtered through a funnel with a folded filter paper to recover the liquid phase. Samples were washed with NaCl solution (0.9%) to obtain two layers. Then, the determination was made using the sulfophospho-vanillin method described by Frings and Dunn (1970) [50]. This method consists of oxidizing cellular lipids to small fragments after chemical digestion with hot concentrated sulfuric acid. After the addition of a solution of vanillin and phosphoric acid, a red complex is formed, and was read on a JASCO CRT-400 spectrophotometer at 530 nm.

Glycogen levels from the midgut gland were quantified according to Lo et al. (1970) [51] with modification. In a glass tube one half milliliter of 30% KOH saturated with Na_2_SO_4_ was added to the sample (weighing approximately 35–50 mg). The above tubes with the screw cap on were put in a boiling water bath for 30 min, and then the tubes were removed from de boiling water bath and cooled on ice. One milliliter of ethanol 96° was added to precipitate the glycogen. The samples were placed on ice for 30 min and then were centrifuged (ROLCO 2036) at 4500 rpm for 10 min. The glycogen precipitates were then dissolved in 1 mL of distilled water. An aliquot of 300 μL of the above glycogen solution was brought to a sample volume of 1 mL by addition of distilled water. One milliliter of 8% phenol solution was added to the above and then, 5 mL of H_2_SO_4_ were added rapidly. The tubes were then allowed to stand for 10 min. They were shaken and placed for 10–20 min in a water bath at 30 min, before readings were taken. The absorption spectrum was read at 490 nm and the standard solution was prepared with rabbit glycogen (5 mg/mL).

Each midgut gland (100–150 mg) was homogenized in cold Tris-HCl (120 mM, pH 7.5, 1:4 w/v) with a potter homogenizer [52]. After centrifugation at 10000 x *g* for 30 min at 4°C [53] the lipid layer fraction was removed and the supernatant (enzyme extract) was stored at -80°C until used as enzyme extract. The total soluble protein was evaluated with Coomassie blue dye method according to Bradford (1976) [54] using bovine serum albumin (Sigma A2058) as standard.

The TBARS level was measured according to Buege and Aust (1978) [55], by the formation of thiobarbituric acid reactive substances (TBARS). Enzyme extract were added to the reaction mixture (trichloroacetic acid 15% (w/v), 2-thiobarbituric acid 0.375% (w/v), and butylhydroxytoluene 0.147 mM) in a ratio of 1:5 (v/v). The mixture was vigorously shaked, maintained in boiling water for 60 min, and immediately cooled at 5°C for 5 min [56]. Then it was centrifuged at 5000 x g for 10 min, and the supernatant was measured spectrophotometrically at 535 nm.

The PO level was evaluated according to Reznick and Packer (1994)[57], by detecting the formation of protein hydrazones as a result of the reaction of dinitrophenyl hydrazine (DNPH) with protein carbonyls. Some minor modifications were performed to the original protocol [58]; briefly, after the protein hydrazone formation, they were precipitated using TCA 30% [59], and then washed 3 times with ethanol: ethyl acetate (1:1 v/v). After the final wash, the protein was solubilized in 1 mL of urea (6 M in 20 mM potassium phosphate buffer, pH 2.5) instead of guanidine hydrochloride. To speed up the solubilization process, the samples were incubated at 37°C in a water bath for 60 min. The final solution was centrifuged to remove any insoluble material. The carbonyl content was calculated from the absorbance measurement at 375 nm, using an absorption coefficient e = 22,000/ M*cm.

The CAT activity was measured by the method of Aebi (1984) [60]. The reaction mixture contained 50 mM phosphate buffer (pH 7.0) and 3 mM H_2_O_2_, and it was recorded spectrophotometrically at 240 nm. One unit of CAT was defined as 1 nmol of H_2_O_2_ degraded per minute per milligram of protein.

Total proteinase activity was assayed using 1% azocasein as the substrate in 50 mM Tris HCl, pH 7.5 [18]. One proteinase unit was defined as the amount of enzyme required to increase the optical density by 0.01 OD units at 440 nm [61]. Determinations were run in triplicates.

Lipase activity of each enzyme extract was determined according to Versaw et al. (1989) [62]. The assay mixture consisted of sodium taurocholate 100 mM, buffer TRIS HCl 50 mM, pH 7.5 and the enzyme extract. After incubation (25°C for 5 min), the substrate β-naphthylcaprylate (Goldbio N-100) dissolved in dimethyl sulfoxide (DMSO) was added to the mix. The mixture was incubated at 25°C for an additional 30 min before adding 20 μL Fast Blue BB (100 mM in DMSO). The reaction was stopped with trichloroacetic acid (TCA) (0.72 N), and clarified with ethyl acetate:ethanol (1:1 v/v). Absorbance was recorded at 550 nm. One lipase unit was defined as the amount of enzyme required to cause an increase of 0.01 OD units at 550 nm [61]. Determinations were run in triplicates. Percentual residual lipase activity in each assayed time was calculated as the percentage of activity remaining in starved animals considering the enzymatic activity of fed animals as 100%.

The reduced glutathione (GSH) level was determined by the method of Moron et al. (1979) [63]. Digestive glands (20–30 mg) were homogenized (1:10) in EDTA 0.02 M. After deproteinization with trichloroacetic acid (TCA 50%), free endogenous GSH was determined using 0.5 mM 5,5-dithiobis-2-nitrobenzoic acid (DTNB). The absorbance was read at 412 nm. GSH was used as standard to calculate nmol/mg of wet tissue.

### Statistical analysis

Data are presented as mean± standard error. The statistical analysis was performed among treatments (F, S and SF) at each time (15, 30, 50 and 80 days). The independence between molting and treatments was tested using the Chi-square test of independence [38, 64]. Data from the specific growth rate, hepatosomatic index, soluble protein, lipid peroxidation, protein oxidation, reduced glutathione, glycogen and total lipids reserves and catalase, lipase, and proteinase activities were analyzed using Generalized Linear Mixed Models (GLMMs) with the statistical program R and the GLMMs package [15, 65], including Fed, Starved and Starved-Fed treatments as fixed factors. The significance level was set at 0.05.

## Results

**Fig 2:**
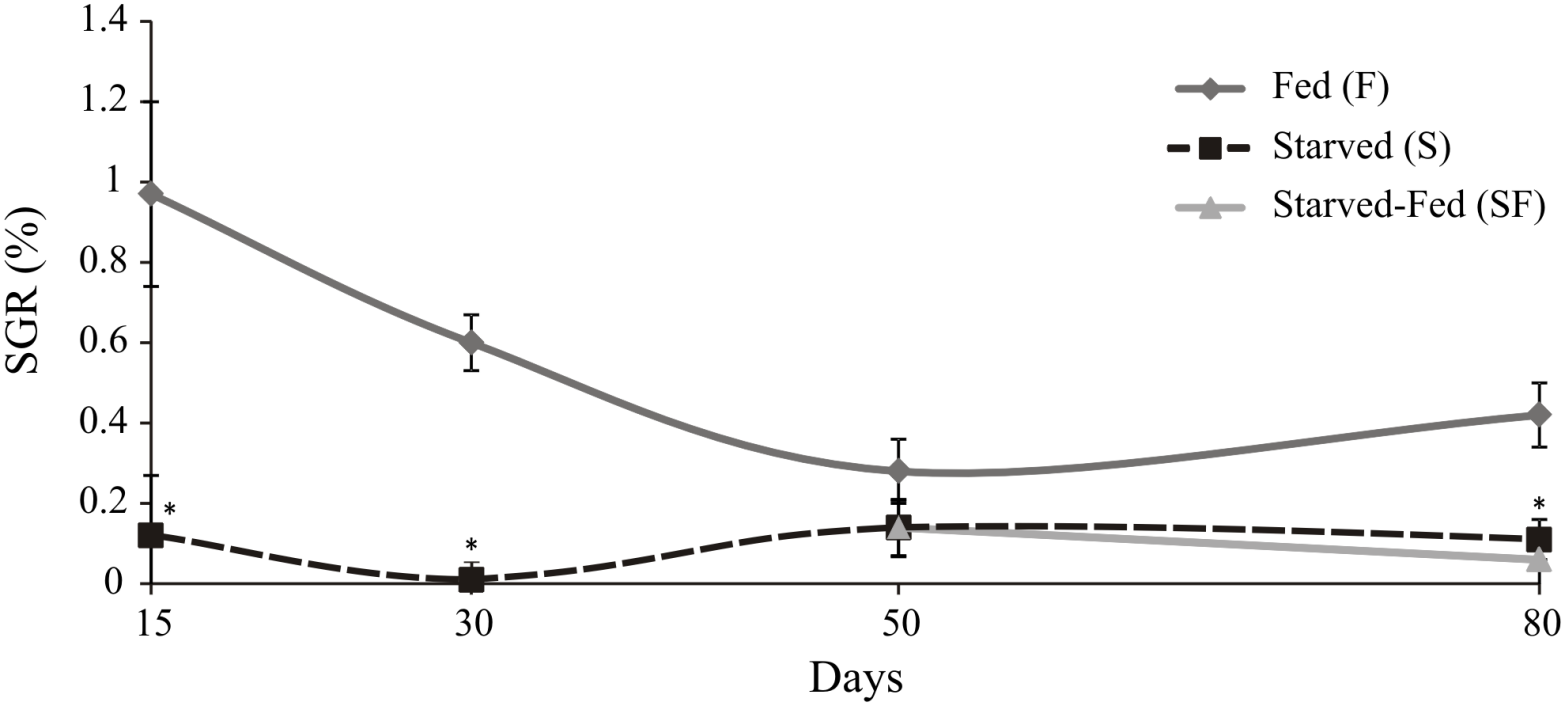
*Cherax quadricarinatus* juveniles’ specific growth rate after starvation and posterior feeding. The statistical analysis was done among treatments (F, S and SF) at each experimental time (15, 30, 50, and 80 days). * indicates statistically significant differences (p<0.05).

The hepatosomatic indexes, molts and soluble proteins are shown in Table 1. Starved crayfish had a lower HSI value (p<0.05) and lower number of molts (p<0.05) than fed animals during the experimental time. Soluble protein levels of starved animals were lower than fed crayfish (p<0.05) only at day 30. The specific growth rate showed a significant lower value (p<0.05) in juveniles under starvation at 15, 30 and 80 days compared to F (Fig 2). The animals of S and SF groups recorded lower SGR (p<0.05) than F group at day 80.

**Table 1:** Hepatosomatic index, number of molts and soluble protein in *Cherax quadricarinatus* juveniles after starvation and posterior feeding.

Treatment	Days	HIS (%)	Molts	Soluble Protein (mg/mL)
**Initial**	0	6.93±0.28	----------	11.79±0.57
**Fed**	15	7.25±0.26	17	11.99±0.42
	30	6.75±0.26	7	12.43±0.43
	50	6.68±0.29	7	12.37±0.41
	80	6.86±0.39	9	9.52±0.57
**Starved**	15	5.16±0.24 *	5 *	10.82±0.50
	30	4.25±0.20 *	1 *	10.65±0.52 *
	50	4.48±0.23 *	0 *	11.80±0.40
	80	4.61±0.22 *	0 *	8.05±0.52
**Fasted-Fed**	80	7.07±0.38	9	9.09±0.47

The statistical analysis was done among treatments at each experimental time.

* indicates significant differences (p<0.05).

### Energetic reserves, oxidative stress and digestive enzymes activity

**Fig 3:**
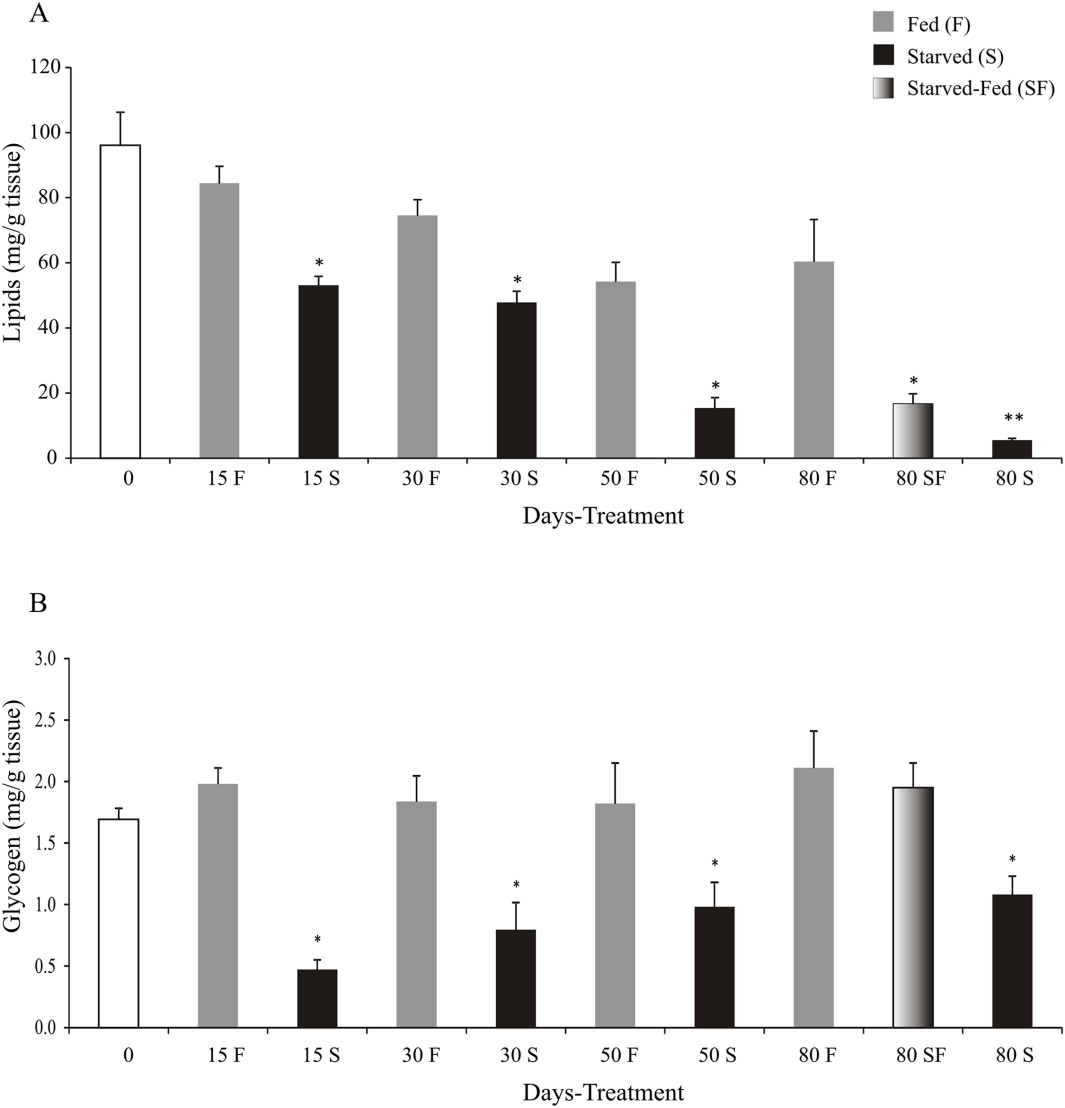
Lipid and glycogen levels of *Cherax quadricarinatus* juveniles after starvation and posterior feeding. The statistical analysis was done among treatments (F, S and SF) at each experimental time (15, 30, 50, and 80 days). * indicates statistically significant differences (p<0.05).

Starved juveniles had lower levels (p<0.05) of lipids and glycogen than control group, at 15, 30, 50 and 80 days. At day 80, crayfish of SF group had lower lipids (p<0.05) and similar glycogen levels (p>0.05) with respect to F group (Fig 3A and 3B).

Lipid peroxidation, protein oxidation and catalase activity are shown in Table 2. No statistical differences (p>0.05) were found in the TBARS, PO and CAT among treatments.

**Table 2:** *Cherax quadricarinatus* juveniles´ lipid peroxidation and protein oxidation levels and catalase activity after starvation and posterior feeding.

Treatment	Days	TBARS (nmol/mg wet weigth)	OP [carbonyl (nmol/mg protein)]	CAT (nmol/mg protein)
**Initial**	0	102.46±5.34	8.33±0.52	2.14±0,53
**Fed**	15	91.53±6.45	8.57±0.53	2.14±0,30
	30	90.56±5.44	8.42±0.59	2.59±0,48
	50	78.94±6.21	7.53±0.68	2.29±0,65
	80	106.98±15.23	8.58±1.13	2.25±0,32
**Starved**	15	86.01±6.79	9.07±0.81	2.18±0,85
	30	99.11±5.74	8.87±0.79	2.84±0,53
	50	90.47±6.55	8.14±0.93	1.45±0,41
	80	83.70±15.23	11.91±1.70	2.16±0,47
**Fasted-Fed**	80	104.64±15.23	9.15±1.82	2.56±0,26

TBARS: lipid peroxidation; PO: protein oxidation and CAT: catalase activity.

The statistical analysis was done among treatments at each experimental time.

**Fig 4:**
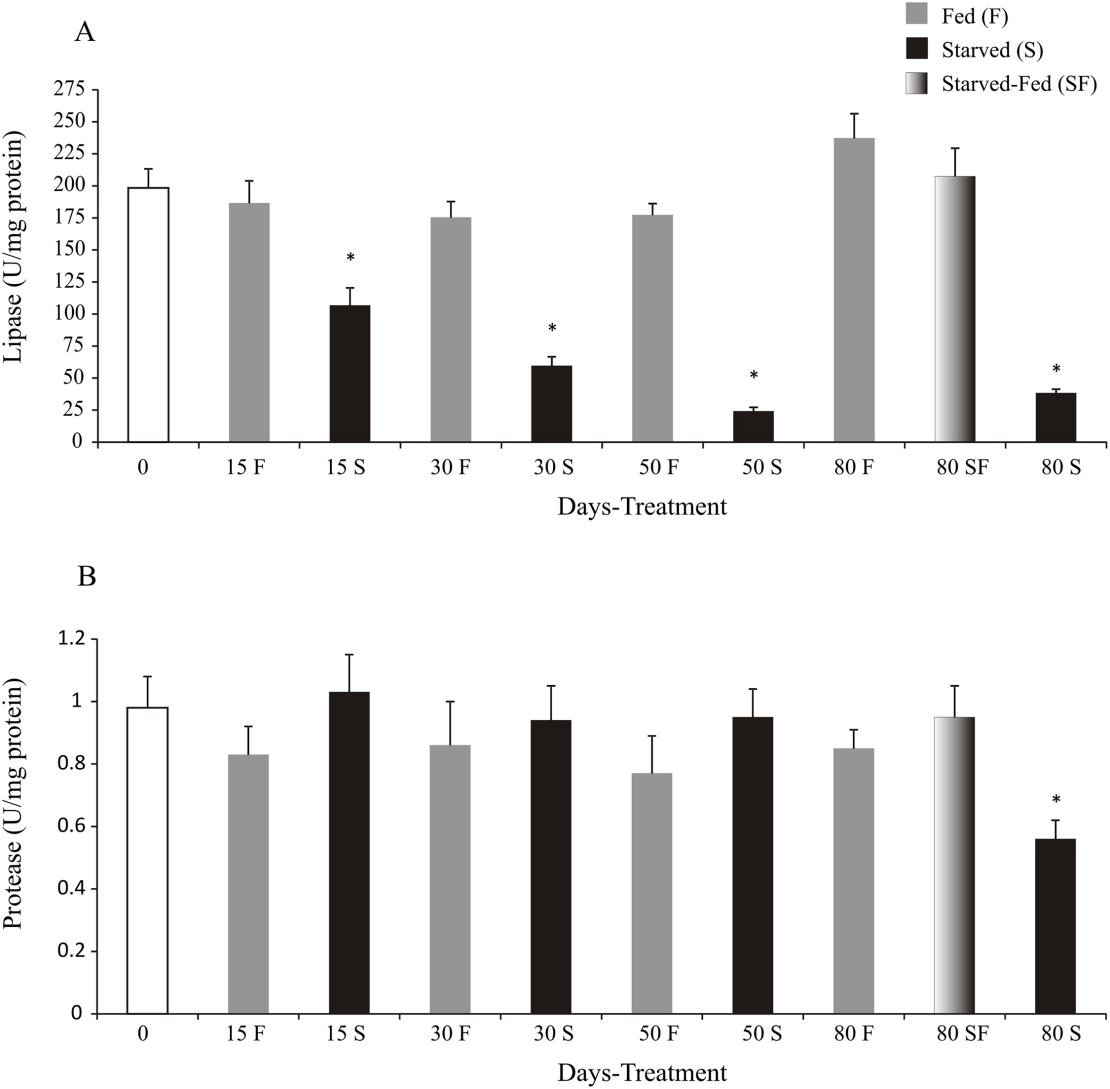
*Cherax quadricarinatus* juveniles´ protease and lipase activities after long starvation and posterior feeding. The statistical analysis was done among treatments (F, S and SF) at each experimental time (15, 30, 50, and 80 days). * indicates statistically significant differences (p<0.05).

After 80 days of fasting, digestive lipase activity decreased in starved juveniles with respect to fed animals, reaching a minimum activity at day 50 and 80 (24.20±2.92 and 38.27±3.14 U/mg protein, respectively) (Fig 4A). Also, the animals of SF group recovered lipase activity at similar values of F group (207.44±22.02 and 237±19.19 U/mg protein, respectively) (Fig 4A). The residual digestive lipase activity of starved juveniles was 57.27%, 33.98%, 13.65% and 16.14% at T15, T30, T50 and T80 respectively.

Protease activity decreased in starved animals with respect to F and SF treatments at day 80 (p<0.05) (Fig 4B).

**Fig 5:**
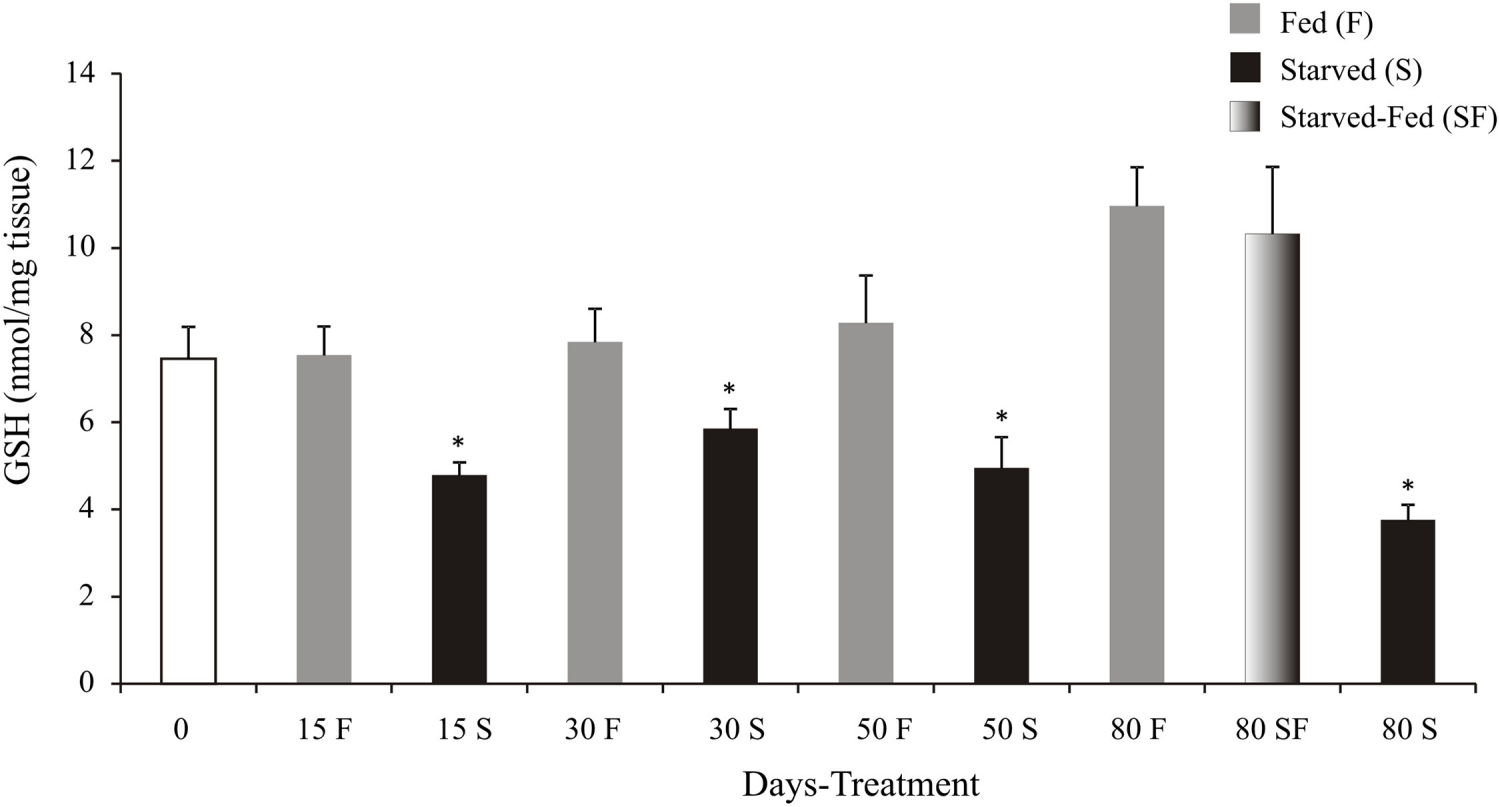
*Cherax quadricarinatus* juveniles´ reduced glutathione levels (GSH) after long starvation and posterior feeding. The statistical analysis was performed among treatments (F, S and SF) at each experimental time (15, 30, 50, and 80 days). * indicates significant differences (p<0.05).

In starved animals the glutathione levels had lower values (p<0.05) than fed animals at all-time tested. At day 80, SF group had similar GSH levels (p>0.05) with respect to F group (Fig 5).

**Fig 6:**
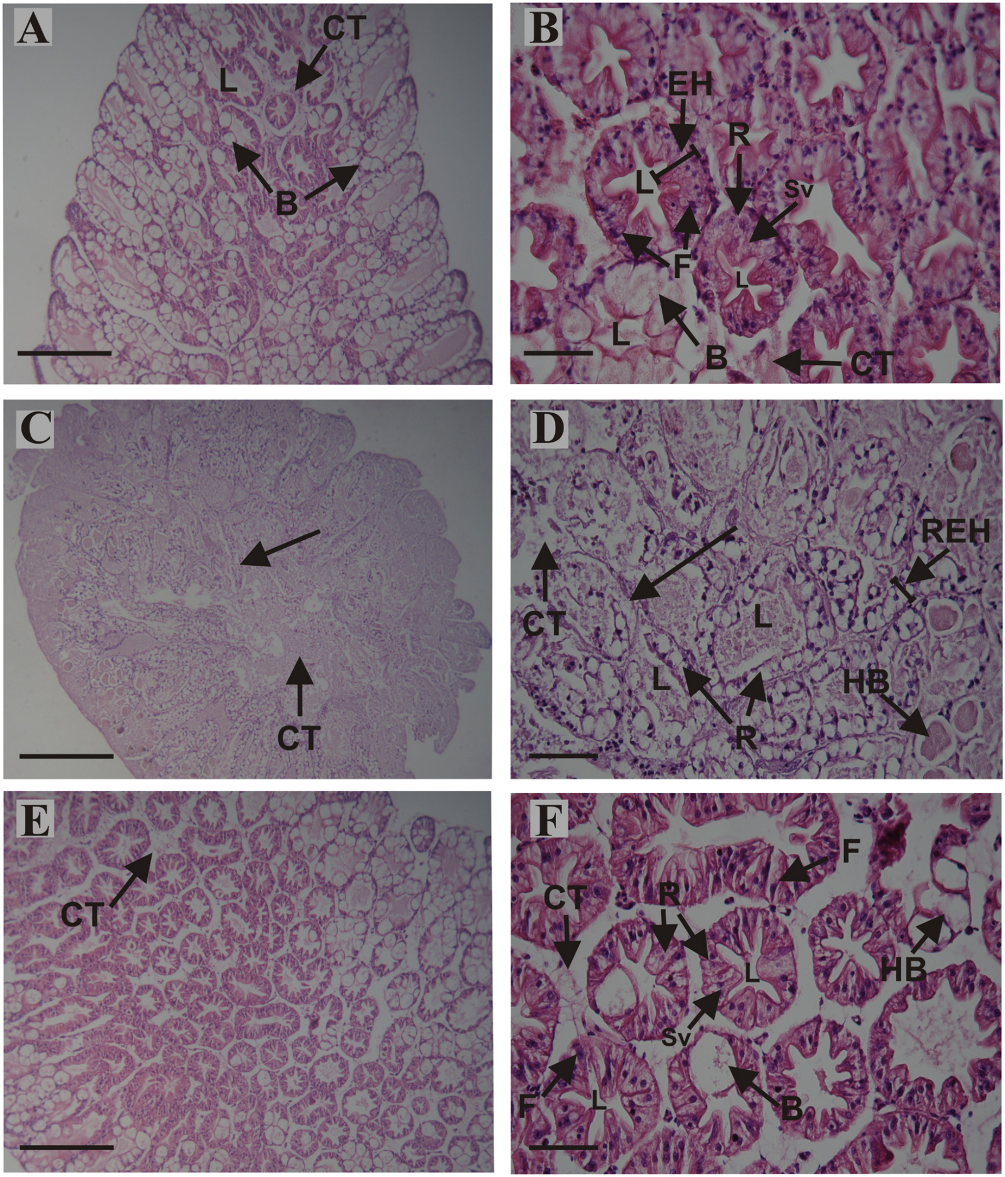
Histological sections of the midgut gland from juveniles of *Cherax quadricarinatus* continuously fed (A, B), abnormalities after long-term starvation (C, D), and abnormalities after long-term starvation and posterior feeding (E, F). (A, C, E): longitudinal section of the apical portion of the tubules; (B, D, F): cross sections of the tubules. (A) and (B): midgut gland structure, connective tissue (CT), epithelial height (EH), F cell, B cell and R cell with small vacuoles (Sv) of fed animals was preserved. (C): structural loss (arrow) and disorganization of connective tissue. (D): undifferentiated epithelium (arrow), partial disorganization of connective tissue (CT), reduction in epithelial height (REH), hypertrophy of B cells (HB), R cells without small vacuoles, scarce differentiation between B and R cells, loss of cell boundaries, reduction in the number of F cells *per* tubule, and loss of the typical star-shaped tubular lumen. (E): partial disorganization of connective tissue. (F): hypertrophy of B cells, R cells with some small vacuoles (Sv), and reduction in the number of F cells *per* tubule. Scale bars: (A, C, E) = 500 μm, (B, D, F) = 100 μm. B: B cell; L: lumen of the tubule; R: R cell.

**Fig 7:**
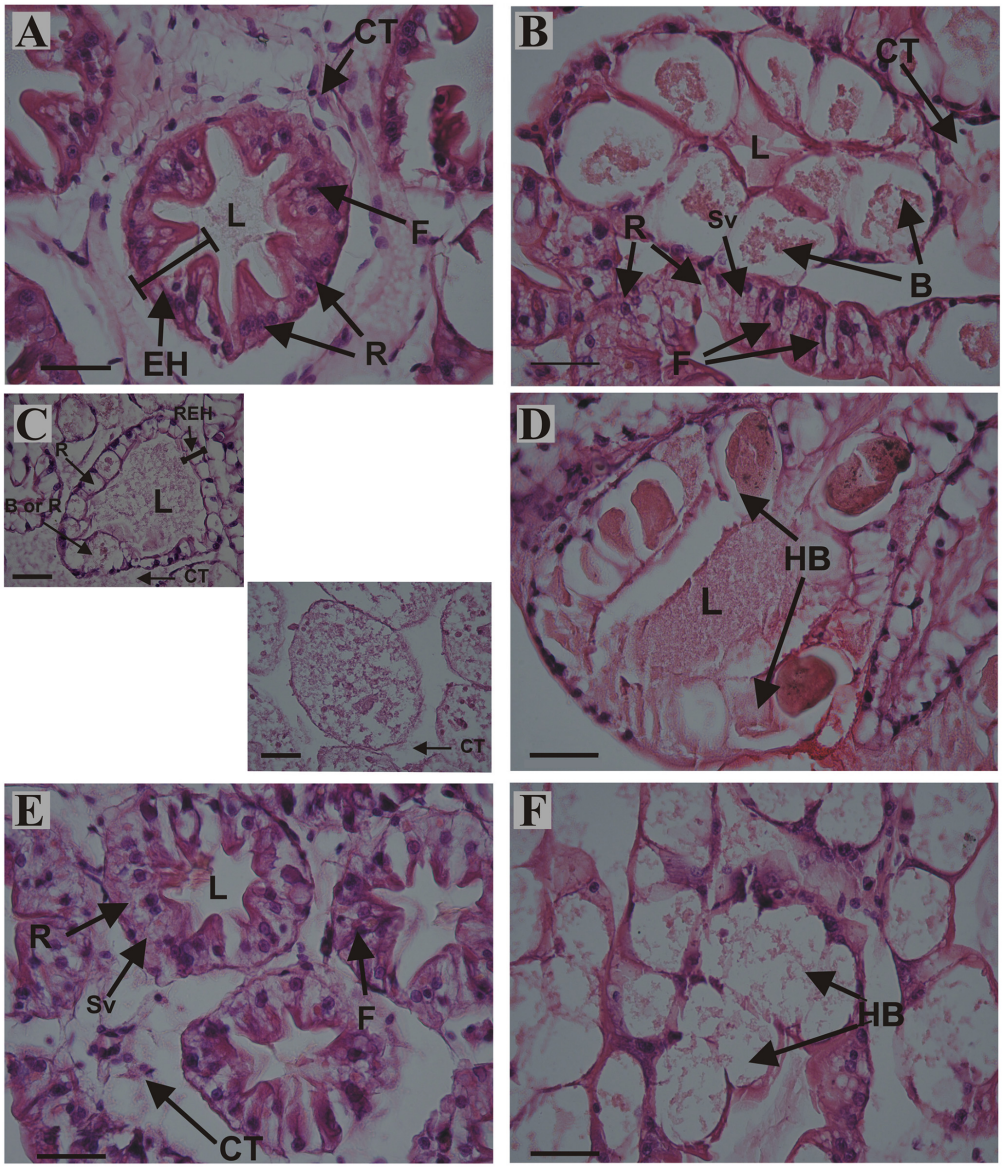
Detail of histological sections of the midgut gland from juveniles of *Cherax quadricarinatus* continuously fed (A, B), abnormalities after long-term starvation (C, D), and abnormalities after long-term starvation and posterior feeding (E, F). (A-F): cross sections of the tubules. (A) and (B): midgut gland structure, connective tissue (CT), epithelial height (EH), F cell, B cell and R cell with small vacuoles (Sv) of fed animals was preserved. (C): undifferentiated epithelium, R cells without small vacuoles, scarce differentiation between B and R cells, partial disorganization of connective tissue (CT), and reduction in epithelial height (REH). (D): hypertrophy of B cells (HB). (E): R cells with some small vacuoles (Sv), partial disorganization of connective tissue (CT), and reduction in the number of F cells *per* tubule. (F): hypertrophy of B cells (HB). Scale bars: 50 μm. B: B cell; L: lumen of the tubule; R: R cell.

Histological observations revealed in the midgut gland structural differences among treatments at day 80 (Figs 6 and 7). The structure of the midgut gland of *C*. *quadricarinatus* resembles that of other decapod crustaceans. It is composed of numerous blind and tubules with four main cell types, namely, E, F, B and R cells (Figs 6A, 6B, 7A and 7B) [11, 12, 66–68]. The midgut gland structure of fed animals was preserved (Figs 6A, 6B, 7A and 7B). After 80 days of starvation, the main changes in the midgut gland of juveniles were: structural loss and disorganization of the tubules; total disorganization of connective tissue; notable reduction in epithelial height (Figs 6C, 6D and 7C); hypertrophy of B cells with one or more large vacuoles, tending to coalesce into single larger ones (Fig 7D); R cells without small vacuoles (Figs 6D and 7C); scarce differentiation between B and R cells (Fig 7C); loss of cell boundaries (arrow) (Fig 6D); reduction in the number of F cells *per* tubule (Fig 6D), and loss of the typical star-shaped tubular lumen (Figs 6D, 7C and 7D). After 30 days of feeding SF animals exhibited a striking recovery (Figs 6E, 6F, 7E and 7F). The main changes respects to F group were: partial disorganization of connective tissue (Figs 6E, 6F and 7E); hypertrophy of B cells (Fig 7F); R cells with some small vacuoles and reduction in the number of F cells *per* tubule (Figs 6F and 7E).

## Discussion

The midgut gland of *Cherax quadricarinatus* showed a clear response to long-term starvation and recovery according to multiple parameters as HSI, histology, molting, SGR, energetic reserves, GSH levels and digestive enzymes.

The midgut gland was severely affected by starvation, and as a consequence the HSI decreased at day 15 in starved animals. This result agrees with previous studies performed in shrimp, crabs and crayfish [16, 19, 21, 29, 33, 69]. In our results, the animals fed for 30 days after starvation, recovered fully the midgut gland weight. Besides, the histological analysis of the starved animals allowed observing several signs of structural changes as well as structural disorganization and loss of the digestive gland tubules, along with R cells without vacuoles, hypertrophied B cells, little differentiation between R and B cells, reduction in the number of F cells, waste of cell boundaries, and loss of “star-shaped” tubular lumen. Most of these alterations have also been observed in early (first free-living stage, JIII, 20 mg approximately), and later (1 g,) juveniles of *C*. *quadricarinatus* under different starvation periods [11, 12].

The long-term starvation caused inhibition of the molting process, and posterior feeding demonstrated that this process is reversible, namely, the persistence of molting capacity. Juveniles of *C*. *quadricarinatus* tolerate long starvation periods, saving energy from exuvia. The inhibition of molting was observed in other studies carried out in shrimp and crayfish [16, 19, 29, 31, 70]. In crustaceans, the molting process is a consequence of growth; this was reflected in the lack of variation of SGR in the starved juveniles, which is in agreement with previous studies [11, 12]. The juveniles fed after 50 days of starvation, did not recover the SGR index. Additionally, in the starved animals the locomotion activity decreased and the color pleon was yellowish (personal observation) in accordance with Calvo et al. (2013) [21] for younger crayfish.

**Fig 8:**
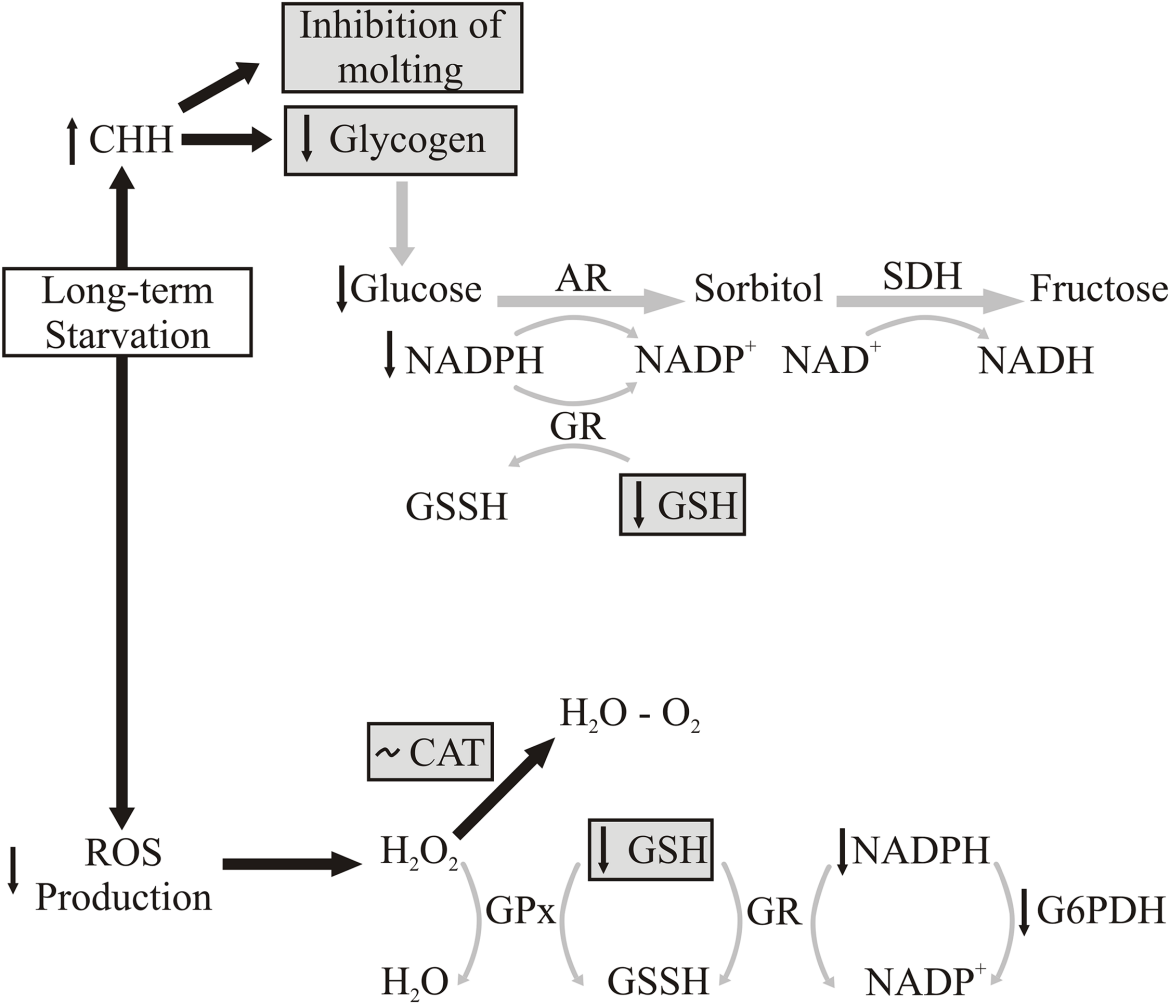
Long-term starvation and possible modification in the regulation diagrams proposed by Fanjul-Moles and Gonsebatt (2012) [22] and Hermes-Lima (2004) [25] in environmental stress situation and ROS production. Black and grey arrows indicate active and inactive pathways respectively. A: Gray boxes indicate the parameters were recorded in the present study. AR: aldose reductase; CAT: catasase; CHH crustacean hyperglycemic hormone; GPx: glutathione peroxidase; GR: glutathione reductase; GSSH: oxidized glutathione; GSH: reduce glutathione; G6PDH: glucose-6-phosphate dehydrogenase; NADH: reduced nicotinamide adenine dinucleotide; NADPH: nicotinamide adenine dinucleotide phosphate; ROS: reactive oxygen species; SDH: sorbitol dehydrogenase.

As a consequence of the hormonal control of metabolism, there is an interrelation among metabolism, endocrine regulation, and oxidative status through the animal´s life cycle [22]. For example, the presence of environmental stressors such as temperature hypoxia and salinity, the actions of crustacean hyperglycemic hormone (CHH) provoke the inhibition molting. Another function of CHH is synthesis and degradation regulation of glycogen across the molt cycle [22] for obtain energy from glycogen reserves by glycogenolysis, and inhibit of molt cycle. Fanjul-Moles and Gonsebatt (2012) [22] suggested that increased glucose levels elicit a rise in ROS production due to the higher flux of reducing equivalents (reduced nicotinamide adenine dinucleotide—NADH) into the mitochondrial electron transport chain [22, 71, 72]. Besides, has been postulated that the pathway of reduction of glucose to sorbitol expends NADPH, and the consumption of NADPH through of regenerating reduced glutathione (GSH) from oxidized glutathione (GSSG) can aggravate intracellular oxidative stress. Based on the previous description and our results during long-term starvation in *C*. *quadricarinatus*, we conceived a possible modification in the regulation diagrams developed by Fanjul-Moles and Gonsebatt (2012) [22] and Hermes-Lima (2004) [25], for environmental stress situation and ROS production that is summarized in Fig 8.

The long-term food restriction (an extremely stressful environmental condition) would cause an increase in CHH, which agrees with our results observed in molting inhibition. The glycogen level, needed to obtain energy during starvation, was low as a consequence of CHH action at day 15. Besides, glucose levels would also decrease, so the Sorbitol pathway would be inactive, with concomitant reductions in NADPH and GSH levels [glutathione reaction, which catalyzes by glutathione reductase (GR)]. According to this pathway, the starved animals exhibited lower GSH levels than fed juveniles during the all- time assayed (Fig 8).

On the another hand, Morales et al. (2004) [23] working in starved fish (*Dentex dentex*) during five weeks demonstrated that glucose-6-phosphate dehydrogenase (G6PDH) could be the key in redox state maintenance of cells, and in regulating the antioxidant defenses through the control of NADPH generation. A reduction of NADPH provokes low GR activity, and this affect GSH regeneration from GSSG. During starved situation has been demonstrated that a reduction in the GSH recycling rate and depletion of endogenous GSH pool [23, 73–77]. This pathway acts in response to H_2_O_2_ elimination through CAT and GPx activities. Furthermore, our results support the concept that this pathway would not be acting (in addition to GSH levels), because the CAT activity was similar among treatments (Fig 8). This result agrees with previous studies which reported that starvation did not induce change in the CAT activity of the rainbow trout´s liver [78]. Other result of the present study would support the notion that the ROS are not generated during a long starvation since no differences were observed in OP and TBARS levels among treatments. To conclude, the long starvation not causes overproduction of glucose (through CHH from glycogen) and ROS in *C*. *quadricarinatus*. When juveniles were fed after a long starvation period, they recovered glycogen and GSH levels; so the Sorbitol pathway became activated, and ROS were not generated.

Another result from our study showed that proteins were not affected by long-term starvation and posterior feeding. This would be indicating that *C*. *quadricarinatus* did not use protein as a primary source of energy during long food restriction. This result disagree with previous studies in shrimps and crabs: *Hemigrapsus nudus* [79], *Marsupenaeus japonicus* [16], *Penaeus esculentus* [80], *Litopenaeus vannamei* [29] and *Lithodes santolla* [33], species that use protein as the main energy source.

This study demonstrates that the glycogen levels decreased quickly in starved animals, and the levels were completely restored after posterior feeding for 30 days. Our result agrees with Mendez and Wieser (1993) [81] in juveniles of *Rutilus rutilus* fish. This was proposed as a strategy to rapidly store food energy, which may be used later for the synthesis of body materials [21].

With respect to lipids, they were gradually consumed as the days of food deprivation increased, and posterior feeding was not sufficient to fully restore the lipid reserves. Therefore, according to our results when the juveniles of *C*. *quadricarinatus* are starved, they first use the glycogen and lipid reserves, leaving the energy from proteins unused. In *C*. *destructor*, Jones and Obst (2000) [1] showed that lipids were mobilized after protein and carbohydrate reserves.

In starved juveniles of *C*. *quadricarinatus*, the digestive lipase activity decreased as starvation time increased. This result agrees with previous studies in the same species. Yudkovski et al. (2007) [82] demonstrated that lipase transcripts diminish in the midgut gland during non-feeding stages and Calvo et al. (2013) [21] found low levels of lipase activity after a 50 day starvation, suggesting that lipase is not synthesized when food is not available. Sacristán et al. (2014) [15] showed that the lipase activity recorded in the animals fasted for 16 days was lower than the animals with food deprivation for 48 hs. Furthermore, Rivera-Pérez and García-Carreño (2011) [83] studying the effect of starvation on the expression of transcripts of lipase in *L*. *vannamei*, showed that there are two types of lipase, a digestive lipase and an intracellular lipase (lysosomal). The digestive lipase is found exclusively in the digestive gland and is negatively regulated during fasting by the absence of food. Whereas the intracellular lipase is expressed in various tissues (digestive gland, uropods, pleopods, digestive tube, gills, hemocytes, muscle and gonads), and it is positively regulated during starvation, suggesting that it is responsible for lipid mobilization from lipids depots (energy reserves) according to Rivera-Pérez and García-Carreño (2011) [83].

Considering the digestive lipase activity of fed animals as 100% in each time assayed, the residual digestive lipase activity of starved juveniles indicates that the lipase activity decreased by half approximately every 15 days without food, and at days 50 and 80 this activity achieved basal levels. Another result was that the digestive lipase activity returned to similar values as the control group in starved animals with posterior feeding. This response concurs with the hypothesis of digestive lipase activity regulation recently proposed by Sacristan et al. (2014) [15]; they proposed that when there is no food for a long period, the intracellular lipase *de novo* synthesis would be stimulated, and as a consequence, lipids stored as energy reserves would be mobilized. The pathway of digestive lipase synthesis is inhibited, barely staying in basal activity levels. Instead, they proposed that the detection of food presence promotes *de novo* synthesis of digestive lipase. The detection of the presence of food would inhibit the intracellular lipase synthesis pathway thereby stored lipids would not be used as an energy source. Additionally, this study`s outcome of lipid reserve level, agrees with the lipase regulation mentioned above.

The proteinase activity was not affected by starvation until 50 days. Hernández-Cortés et al. (1999) [84] demonstrated in the crayfish *Pacifastacus leniusculus*, the presence of trypsinogen in the digestive gland. Furthermore, Sainz et al. (2004)[85] studying trypsin synthesis and storage as zymogen in fed and fasted animals of *Litopenaeus vannamei*, revealed that trypsinogen is not secreted totally from a single cell (B cell), it appears to be secreted partially as a result of ingestion. Therefore, the significant decrease of protease activity at day 80 could be due to structural loss, as was demonstrated histologically in the midgut gland.

The present study provides new and relevant biological information on physiological responses of crayfish under long-term starvation. According to the whole results of the present research, when the redclaw crayfish *C*. *quadricarinatus* are long-term starved, they do not grow (SGR and no molting), reducing the digestive gland weight (HSI), presenting histological alteration in the midgut gland, using the glycogen and lipid reserves as source energy, reducing digestive lipase activity and GSH levels, and will not be altering the catalase activity. Therefore, these parameters could be used as a tool to analyze the nutritional status of *C*. *quadricarinatus*.
